# Clonally expanded human airway smooth muscle cells exhibit morphological and functional heterogeneity

**DOI:** 10.1186/1465-9921-15-57

**Published:** 2014-05-03

**Authors:** Shailendra R Singh, Charlotte K Billington, Ian Sayers, Ian P Hall

**Affiliations:** 1 Division of Respiratory Medicine, University of Nottingham, Queen’s Medical Centre, NG7 2UH Nottingham, United Kingdom

**Keywords:** Human airway smooth muscle, Clonal expansion, Phenotype, Plasticity, Remodeling

## Abstract

**Background:**

Mesenchyme-derived airway cell populations including airway smooth muscle (ASM) cells, fibroblasts and myofibroblasts play key roles in the pathogenesis of airway inflammation and remodeling. Phenotypic and functional characterisation of these cell populations are confounded by their heterogeneity *in vitro*. It is unclear which mechanisms underlie the creation of these different sub-populations.

The study objectives were to investigate whether ASM cells are capable of clonal expansion and if so (i) what proportion possess this capability and (ii) do clonal populations exhibit variation in terms of morphology, phenotype, proliferation rates and pro-relaxant or pro-contractile signaling pathways.

**Methods:**

Early passage human ASM cells were subjected to single-cell cloning and their doubling time was recorded. Immunocytochemistry was performed to assess localization and levels of markers previously reported to be specifically associated with smooth muscle or fibroblasts. Finally functional assays were used to reveal differences between clonal populations specifically assessing mitogen-induced proliferation and pro-relaxant and pro-contractile signaling pathways.

**Results:**

Our studies provide evidence that a high proportion (58%) of single cells present within early passage human ASM cell cultures have the potential to create expanded cell populations. Despite being clonally-originated, morphological heterogeneity was still evident within these clonal populations as assessed by the range in expression of markers associated with smooth muscle cells. Functional diversity was observed between clonal populations with 10 μM isoproterenol-induced cyclic AMP responses ranging from 1.4 - 5.4 fold cf basal and bradykinin-induced inositol phosphate from 1.8 - 5.2 fold cf basal.

**Conclusion:**

In summary we show for the first time that primary human ASM cells are capable of clonal expansion and that the resulting clonal populations themselves exhibit phenotypic plasticity.

## Background

Human airway smooth muscle (ASM) cells are key players in the development, orchestration and perpetuation of asthma pathology [1]. In addition to forming the muscle mass which causes bronchospasm in asthma exacerbations, human ASM cells also mediate inflammation *via* cytokine release [1]. Increased ASM mass is a feature of the airway remodeling seen in many individuals with chronic asthma, where it is associated with disease severity [2-4]. Increased ASM area has also been shown to correlate with airflow obstruction (likely due to a combination of increased contractility and decreased lumen size) [5].

The mechanisms behind the increase in ASM mass in asthma may involve hyperplasia, hypertrophy, decreased apoptosis, altered migration, increased recruitment of fibrocytes or increased differentiation of mesenchymal stem cells or *via* epithelial-mesenchymal differentiation, or indeed a combination of any or all of the above [6]. Whilst *in vivo* little is known of the clinical relevance of these mechanisms, *in vitro* the ASM cell signaling pathways key to these events have been extensively researched and many pro-proliferative, pro-apoptotic and pro-migratory mediators identified [7]. In addition to these molecules, recent evidence demonstrates the ability of bronchoconstriction itself to induce airway remodeling both in guinea-pigs [8] and humans [9].

It is also important to consider how phenotypic switching of ASM cells could impact on ASM mass. Phenotypic switching or phenotype plasticity refers to the change in an ASM cell classically between a “contractile” (or even “hypercontractile”) and “synthetic” or “proliferative” state [10]. *In vitro* phenotypic plasticity has been demonstrated as being tightly regulated: growth factors, fibronectin, collagen type I, integrins and adhesion molecules are observed to induce a synthetic phenotype whereas serum deprivation, Transforming Growth Factor β (TGF-β) and insulin are observed to induce a contractile phenotype (see [10]).

Given the phenotypic heterogeneity which ASM cells can exhibit *in vitro*[11], we aimed to investigate the clonal origin of populations of cells in ASM in order to determine whether single cells were capable of clonal expansion and also to define how different resulting clones might be from each other and their parental cell in terms of morphology and function. Functional differences were investigated by assessing pro-relaxant and pro-contractile second messenger signaling pathways, namely β_2_-adrenoceptor-mediated cyclic AMP formation and bradykinin B1 and histamine H1 receptor-mediated inositol phosphate production respectively.

This is the first study to show that a significant proportion of single cells present within early human ASM cell cultures have the potential to create expanded cell populations. These clonal cell populations were observed to be morphologically and functionally diverse.

## Methods

### Human airway smooth muscle cell culture

Human airway smooth muscle (ASM) cells were prepared as previously described via enzymatic dispersion from four individuals undergoing thoracotomy [12]. Ethical approval was obtained from the local Nottingham ethical research committee (EC00/165). Cells were cultured in Dulbecco’s modified Eagles media (DMEM) containing 10% fetal calf serum and glutamine (2 mM) and incubated at 37°C in 5% CO_2_. Cells isolated and cultured in this way have been extensively characterised and shown to retain many of the phenotypic properties of freshly isolated airway smooth muscle cells [13-15]. Cells expanded for use in the signaling assays originated from one donor.

### Single cell sorting via Fluorescence-activated cell sorting (FACS)

Early passage (passage 2–4) human ASM cells were grown to ~90% confluency and cultured in 0.5% foetal calf serum (FCS) supplemented DMEM medium for 24 hours. Cells were then exposed to trypsin to place the cells in suspension, pelleted, resuspended in PBS and then subjected to single cell sorting in 96 well culture plates using Coulter Epics Altra Hypersort fluorescence-activated cell sorter system. Using a forward versus side scatter plot the cell sort gating criteria ensured that only cells with similar morphological characteristics were used in this process. Cells were singly-sorted directly into the 96 well plate with each well containing DMEM medium supplemented with 20% FCS and glutamine. Single cell occupancy in each well of 96 well plates was confirmed by thorough microscopic examination and those wells with no cells or more than one cell were marked and not included any further in the experiment. With the exception of the increased percentage of FCS (20%), cells were thereafter cultured using the standard techniques specified above. Growth medium was replaced 7 days after plating and then afterwards every 2 days. As the cells achieved confluency in one well of the 96 well plate (surface area: 0.3 cm^2^), they were trypsinised and subcultured into one well of a 24 well plate (surface area: 1.9 cm^2^) and at this stage were cultured using standard techniques for human ASM cells (DMEM medium supplemented with 10% FCS and 2% glutamine). Once confluency was achieved in one well of a 24 well plate, the cells were subcultured into one well of a 6 well plate (surface area: 9.4 cm^2^) and once this well contained a confluent monolayer cells were subcultured into a 25 cm^2^ culture flask (T25). Throughout this timecourse the times at which confluency was achieved in the diverse vessel sizes was recorded and hence doubling times could be calculated. The values expressed were mean ± Standard Error Mean (SEM) of the doubling time recorded for cells that proliferated.

### Single cell sorting via serial dilution

Early passage (2–4) human ASM cells were cultured to ~90% confluence then grown in medium containing 0.5% FCS then exposed to trypsin to place the cells in suspension. The total cell count in the suspension was determined using a haemocytometer prior to the cell suspension being subjected to serial dilutions until calculations predicted that one cell should be present per 250 μl of DMEM medium (supplemented with 20% FCS and glutamine). Each 250 μl sample (containing one cell according to calculations) was placed into a separate well of a 96 well plate and incubated at 37C, 5% CO_2_. Single cell occupancy in each well of 96 well plates was again confirmed by thorough microscopic examination and those wells with no cells or more than one cell were not included any further in the experiment. Cells were then cultured and doubling times assessed exactly as described above (Single Cell Sorting *via* FACS).

For some analyses, clonal cell populations were grouped based on the time required for the clones to achieve confluency in culture plates in initial experiments: I) Fast Growing clonal populations: Populations achieving confluency in a 25 cm^2^ tissue culture flask in less than 45 days and II) Slow Growing clonal populations: Populations achieving confluency in a 25 cm^2^ tissue culture flask in 45 days or more.

### [^3^H]-Thymidine incorporation in human ASM cells

[^3^H]-Thymidine incorporation in human ASM cells was assessed as previously reported with minor modification [16]. Cells were seeded at 2.5 × 10^4^ cells/ well and grown to subconfluence (70–90%) in 24-well plates were washed and incubated in DMEM containing 0.1% FCS and 2 mM glutamine for 24 h to growth arrest the cells. Platelet derived growth factor (PDGF-BB) at a range of concentrations (20 fg/ml to 20 ng/ml) was added and present in the well for a total of 24 h with [^3^H]-thymidine (1 μCi/well) being added and present for the final 16 h of the incubation. At the end of this period, the supernatant was aspirated, and the cells were washed twice with PBS before being fixed with methanol-glacial acetic acid (3:1) for at least 1 h at room temperature. Two further washes with methanol–water (4:1) were performed before the cells were lysed with 1 ml of 1 M NaOH. Nine hundred microliters of the supernatant were transferred to a scintillation vial along with 10 ml of scintillation fluid (Packard, Meriden, CT) and counted on a LKB scintillation counter (efficiency ∼ 30%), the results being expressed as disintegrations per minute or as a multiple of stimulation over the control value.

Proliferation rates were expressed as mean ± SEM. Donor and passage-matched human ASM cells (passage 9) were referred as the ‘standard’ cell type. Four Fast Growing clonal populations and five Slow Growing clonal populations (as defined above) were used.

### Determination of cyclic AMP accumulation in human ASM cells

Accumulation of [^3^H] cyclic AMP was measured by a modification of a previously described method [16]. In brief, confluent monolayers of cells plated at 2.5 × 10^4^ cells/ well in 24 well plates were labeled with [^3^H]adenine (2 μCi/well) for 2 h in DMEM at 37°C. At the end of this period, the cells were washed three times with 1 ml of Hanks-HEPES buffer and allowed to rewarm to 37°C for 20 min in the presence or absence of a range of concentrations of the β-adrenoceptor agonist isoproterenol (10^−9^ to 10^−5^ M) before the reactions were terminated by the addition of 50 μl of concentrated HCl. The cells were then stored at −20°C. [^3^H] cyclic AMP was determined by column chromatography after the cells were rethawed as previously described [16]. Aliquots of [^14^C] cyclic AMP were added to each sample, and the counts obtained from this recovery marker were used to correct for variations in recovery from each column. In addition, a 100 μl aliquot was taken from each well of the plate after the reactions were stopped and counted for tritium to correct for variations in the number of cells per well.

Triplicate wells were counted for each condition, and the data are expressed as fold change (compared with basal counts). Mean data are presented (±SEM). Donor and passage-matched human ASM cells (passage 9) were referred as the ‘standard’ cell type.

### Determination of Total [^3^H] Inositol Phosphate in human ASM cells

[^3^H] Inositol phosphate formation was determined as described below. Near-confluent cell monolayers in 12 well plates were incubated for 24 h at 37°C with 500 μl of inositol-free DMEM containing [^3^H] myoinositol (47 Ci/mmol) at a concentration of 4 μCi/ml. After loading, cells were washed once with PBS. Inositol-free DMEM containing 10 mM LiCl was added to each well and the cells were incubated for 10 min at 37°C. Cells were then stimulated with a range of concentrations of histamine (10^−9^ to 10^−4^ M) or bradykinin (10^−10^ to 10^−5^ M) for 10 min at 37°C. Reactions were stopped by aspirating medium and adding 0.8 ml of ice-cold 0.4 M perchloric acid. A total of 0.4 ml of 0.72 N KOH/0.6 M KHCO_3_ was added, and the sample was centrifuged to settle the precipitate. The supernatant was applied to 1 ml AG1-X8 (Bio-Rad, Hercules, CA) columns (100 to 200 mesh, formate form), which were washed with 10 ml of 0.1 N formic acid, and total inositol phosphates were eluted with 1.5 M ammonium formate/0.1 N formic acid and counted. Donor and passage-matched human ASM cells (passage 9) were referred as the ‘standard’ cell type.

### Fluorescent immunocytochemistry

Cells were cultured and immunostained using published methods [11]. Primary antibodies and concentrations used were: α-smooth muscle actin, 0.4 μg/ml; fibroblast surface protein, 0.4 μg/ml; aldo-keto reductase 1 (AKR1C3), 11 μg/ml (Sigma-Aldrich, Poole, UK); cathepsin K, 2.5 μg/ml (Abcam, Cambridge, UK); thromboxane synthase, 8 μg/ml (Proteintech, Manchester, UK); α8 integrin, 1 μg/ml (Insight Biotechnology, Wembley, UK). The secondary antibodies and dilutions used were: polyclonal goat anti-mouse antibody labelled with alexafluor 488, 1:250; polyclonal goat anti-rabbit antibody labelled with rhodamine red-X, 1:250 (both from Invitrogen, Paisley, UK). Cell nuclei were stained with DAPI (1 μg/ml, diluted in PBS) and the cells mounted using DAKO fluorescent mounting media (Dakocytomation, Cambridgeshire, UK). Cells were then visualised using 20× and 40× objective lens on an epifluorescent microscope (Nikon, Surrey, UK, Diaphot 300 + Hg lamp) by SPOTCam Advanced software (Image Solutions, Chorley, Lancashire, UK). The published images were recorded by applying background subtraction and keeping a constant exposure time and gain for each antibody to facilitate qualitative comparison.

### Statistical analyses

For all assays, across group differences were analysed by two-way ANOVA. Figures represent mean values ± SEM. Statistical analyses and curve fitting were completed by using GraphPad Prism v5 (GraphPad, San Diego, CA, USA); a p-value < 0.05 was considered significant.

## Results

### Early passage human ASM cells are capable of clonal expansion and exhibit distinct variations in growth rates

**Figure 1:**
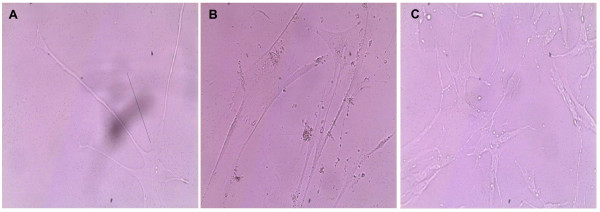
**Clonal expansion of early passage human ASM cells**. Early passage human ASM cells were single sorted by fluorescence-activated cell sorting and subcultured in 20% or 10% FCS containing DMEM medium at 37°C, 5% CO_2_ atmosphere. All images were taken from the same well and used ×40 objective **(A)** Single human ASM cell as seen in culture on day 2 (see black arrow). **(B)** Single human ASM cell proliferating to give rise to a number of cells (day 5). **(C)** Further expansion at day 18.

Initial experiments were performed to determine whether it was possible for single-sorted human ASM cells to survive and proliferate in culture. Following sorting, each well was thoroughly examined microscopically to ensure that no more than one cell was present. Figure 1 shows an image of cell(s) observed within the same well on days 2, 5 and 18.

**Figure 2:**
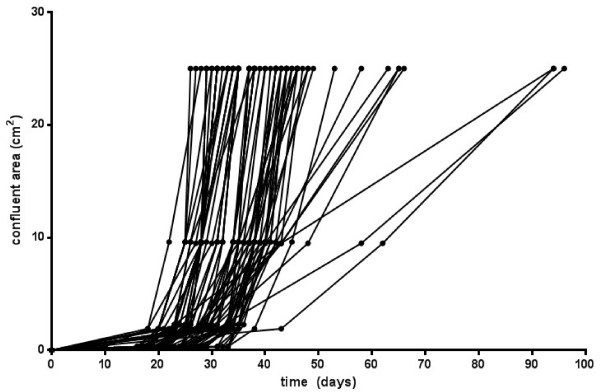
**Proliferation rates of clonally expanded early passage human ASM cells.** Human ASM cells (passage 2–4) were clonally expanded following fluorescence-activated cell sorting or dilution cloning techniques and doubling time of clones expanded from single cell human ASM cultures in four independent experiments was recorded. Data represents the time in which clonal cell populations became confluent over culture vessels with different surface areas.

Overall a single human ASM cell was detected in 130 of the 768 wells seeded (16.9%) with the rest of the wells either demonstrating the presence of more than one human ASM cell or no cell at all. These were therefore excluded from all further analyses. Of the 130 cells which were successfully single-sorted, a total of 76 clones (58%) expanded sufficiently to quantify doubling times (i.e. at least achieved confluency in the original well of the 96 well plate). When doubling times were assessed they were observed to range from 1.1 days to 18.3 days (Figure 2). A further 54 clones (42% of the wells seeded with single cells) did not reach confluency over the time course of each individual experiment (3 months) with the majority of these undergoing senescence.

### Clonally expanded human ASM cell populations exhibit phenotype heterogeneity

To further explore the heterogeneity of cells within a clonal population, cells were passaged further (see methods) giving sufficient numbers for immunocytochemistry and functional assays to be performed. Here to best investigate the phenotypic range, clonal cell populations were grouped based on the time required for the clones to achieve confluency in culture plates in initial experiments (see Figure 2). These were defined as follows: I) Fast Growing clonal populations: Populations achieving confluency in a 25 cm^2^ tissue culture flask in less than 45 days and II) Slow Growing clonal populations: Populations achieving confluency in a 25 cm^2^ tissue culture flask in 45 days or more.

**Figure 3:**
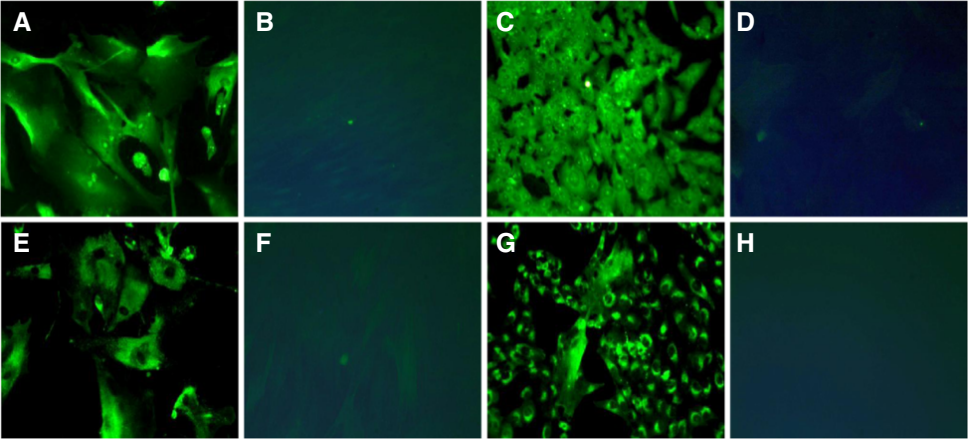
**Immunocytochemical expression of standard mesenchymal cell marker**. Fast and slow growing clonally expanded human ASM cell populations were grown on 8-well chamber glass slides to 80% confluency. Following cell fixation, slow and fast growing clonal populations were immunocytochemically labelled for α-smooth muscle actin (**A** &**C** respectively; 0.4 μg/ml; **B** &**D** corresponding isotype control) and fibroblast surface protein (**E** &**G** respectively; 0.4 μg/ml; **F** &**H** corresponding isotype control). Images were captured with ×20 objective lens using an epifluorescent microscope with matched exposure time and gains. Images are representative of 3 independent experiments.

**Figure 4:**
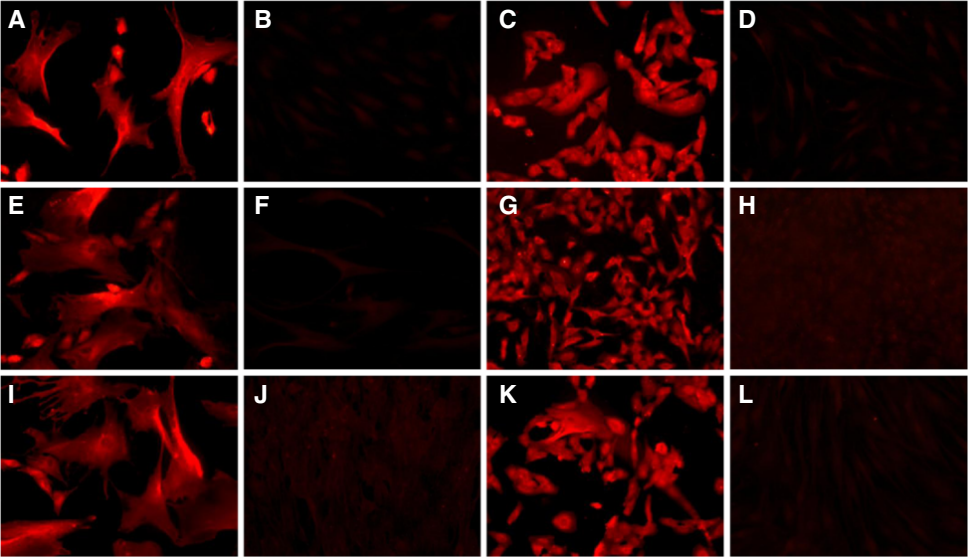
**Immunocytochemical localization of novel mesenchymal cell markers**. Fast and slow growing clonally expanded human ASM cell populations were grown on 8-well chamber glass slides to 80% confluency. Following cell fixation, slow and fast growing clonal populations were immunocytochemically labelled for cathepsin K (**A** &**C** respectively; 2.5 μg/ml; **B** &**D** corresponding isotype control), thromboxane synthase 1 (**E** &**G** respectively; 8 μg/ml; **F** &**H** corresponding isotype control) and α8 integrin (**I** &**K** respectively; 1 μg/ml; **J** &**L** corresponding isotype control). Images were captured with ×20 objective lens using an epifluorescent microscope with matched exposure time and gains. Images are representative of 3 independent experiments.

These studies demonstrated expression of the classical mesenchymal cell phenotypic markers, α-smooth muscle actin (Figure 3A & C) and fibroblast surface protein (FSP) (Figure 3E & G) in both slow- and fast-growing clonal human ASM cell populations. In all cases cells were serum starved to ensure differences were not cell cycle dependent. The expression profile of these characteristic markers revealed some qualitative differences (see Table 1) with counter-intuitively, the slow-growing populations demonstrating increased levels of FSP and decreased levels of α- smooth muscle actin when compared with the fast-growing populations. We also investigated the expression of the novel mesenchymal cell markers identified in our previous work [11] in these clonal populations. Aldo-keto reductase 1 C3 (AKR1C3) expression could not be observed at detectable levels in either the fast- or slow-growing clonal cell populations (figure not shown). However, both clonal cell populations studied expressed cathepsin K (Figure 4A & C), TBXAS1 (thromboxane synthase 1, Figure 4E & G), and α8-integrin (Figure 4I & K) with no obvious qualitative difference in their expression levels or cellular localisation (see Table 1).

**Table 1:** Immunofluorescence against known phenotypic markers performed on fast and slow growing clonal populations

**Phenotype markers**	**Slow growing clonal populations**	**Fast growing clonal populations**
α-smooth muscle actin	++	+++
Fibroblast surface protein	++	+
Cathepsin K	+	++
Thromboxane synthase	++	++
α8 integrin	++	++

Staining intensity was categorized into 3 grades (+, low staining; ++, moderate staining; +++, intense staining) and recorded by blinded observations. Results represent observations from 3 independent studies.

### Human ASM cell clonal populations show morphological heterogeneity

**Figure 5:**
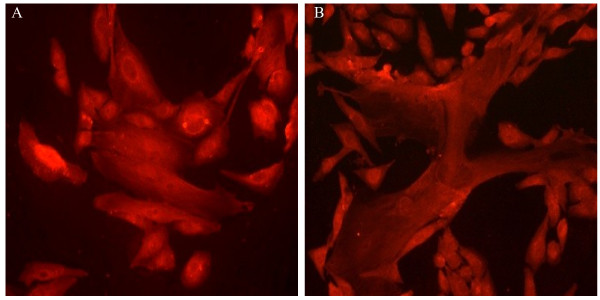
**Morphological characterisation of early passage human ASM cells following single-cell cloning**. Following fluorescence-activated cell sorting based single-cell sorting, early passage human ASM cells were subjected to clonal expansion and sub cultured in 10% foetal calf serum supplemented growth media. Their morphological characteristics were observed and recorded in **(A)** slow-growing clonal populations and **(B)** fast-growing clonal populations at passage 7. Cells are labelled for cathepsin K (2.5 μg/ml). Representative images of isotype controls are shown in Figure 4B & D.

Following clonal expansion, and after repeated sub culturing, variations in cellular morphology were observed within single clonal populations, thus despite all cells within that population originating from a single cell (Figure 5A & B, here immunostained to show cathepsin K expression). Morphological heterogeneity was evident in the form of elongated-spindle shaped, short-spindle shaped or broad-elongated cell populations. Such morphological variations were seen in all clonal populations irrespective of their proliferative potential and are also evident when cells within the same field are compared for Figures 3 and 5.

### PDGF-stimulation induces significant differences in [^3^H] thymidine uptake amongst different human ASM clonal populations

**Figure 6:**
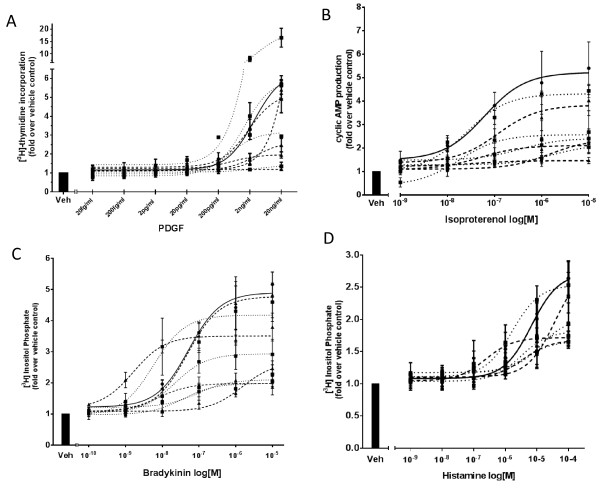
**Altered proliferative and signaling responses in human ASM clonal populations.** In all panels each data point represents mean ± SEM (n = 3). Four Fast Growing clonal populations (•••) and four-five Slow Growing clonal populations (− − −) (as defined in the methods) were used in addition to the standard (not clonally derived) donor population (**─**). **(A)** [^3^H] thymidine incorporation in human ASM clonal populations in response to a range of PDGF-BB concentrations (0.00002 ng/ml to 20 ng/ml) or vehicle alone (Veh). Statistical analyses identifies significant difference in the proliferative response across all the clones (p < 0.001, two-way ANOVA). **(B)** cAMP formation in clonal human ASM populations in response to stimulation with varying concentrations of isoproterenol (10 μM to 1 nM) or vehicle alone (Veh). Significant differences were observed in cAMP formation across all the clonal populations (p < 0.001, two-way ANOVA). **(C)** [^3^H] Inositol Phosphate accumulation following stimulation of human ASM clonal populations with varying concentrations of bradykinin (10 μM to 0.1 nM) or vehicle alone (Veh). Statistical analyses of the data across all the clones showed a significant difference in the production of tritiated inositol phosphates (p < 0.001, two-way ANOVA). **(D)** [^3^H] Inositol Phosphate accumulation following stimulation of human ASM clonal populations with a range of histamine doses (100 μM to 1nM) or vehicle alone (Veh). Significant difference was observed in the total inositol phosphate accumulation across all the clonal groups following statistical analysis (p = 0.0023, two-way ANOVA).

To further quantify the differences in proliferative capacity observed in the clonal populations [^3^H] thymidine uptake was assessed in response to the potent human ASM cell mitogen, Platelet-Derived Growth Factor (PDGF) at a concentration previously observed to be maximal in terms of human ASM cell proliferation (20 ng/ml) [17]. Twenty four hour exposure to PDGF-BB induced a range from 1.19 ± 0.06 to 16.49 ± 4.63 (mean ± SEM) fold increase over basal in [^3^H] thymidine incorporation (Figure 6A). For comparative purposes unsorted donor-matched cells were grown to the same confluence and used as a benchmark “standard” for responses. [^3^H] thymidine uptake in human ASMs grown under standard culture conditions (i.e. not subjected to single cell sorting or clonal expansion) following stimulation with PDGF (20 ng/ml) for 24 hours was observed to be 5.74 ± 0.19 (mean ± SEM) fold over the control.

The average [^3^H] thymidine uptake observed in all fast-growing clonal populations in response to PDGF stimulation (20 ng/ml) for 24 hours was 8.22 ± 2.4 (n = 4, mean ± SEM) fold over control and that observed in slow-growing clones was 3.27 ± 0.3 (n = 3, mean ± SEM) fold over control. However this difference was not statistically significant due to the diverse range of inter-clonal population responses observed even within these groups. (p = 0.1). Only two clones classified as fast-growing had a higher [^3^H] thymidine uptake compared to the standard. Overall, there was a significant difference in the growth rates across all the clones (p < 0.001 by two-way ANOVA).

### Altered Isoproterenol-induced cyclic AMP production in human ASM clones

In order to examine potential functional heterogeneity between cell populations expanded from single human ASM progenitors, we chose to study signaling responses in two physiologically and clinically important pathways present in these cells: β_2_-adrenoceptor mediated signaling constituting the major pro-relaxant pathway in ASM cells and IP3 production used as a functional readout for pro-contractile Gq-coupled receptor activation.

First, we investigated cyclic AMP production in all the cell clones following stimulation with the β-adrenoceptor agonist, isoproterenol at a range of different concentrations (10 μM to 1 nM). Figure 6B demonstrates the isoproterenol-induced dose-dependent increase in cyclic AMP production observed in both clonal populations and the unsorted, donor-matched “standard” cells. The maximum cyclic AMP accumulation observed was recorded in data from a single Fast Growing clonal population (4.4 ± 0.3 fold c.f. basal) in response to exposure to 10 μM isoproterenol. However this effect was lower compared to the unsorted, donor-matched ‘standard’ cell response achieved at the same concentration of isoproterenol (5.4 ± 1.12, Figure 6B). The average cyclic AMP production recorded in all fast-growing clones was 2.8 ± 0.1 (n = 3) and 2.74 ± 0.18 (n = 3) in all slow-growing clonal cells on stimulation with 10 μM of isoproterenol. Statistical analyses by two-way ANOVA demonstrated a significant difference in cyclic AMP production across all the clones thus the clones exhibited diverse signalling capacities (p < 0.001). However the variation observed in cyclic AMP accumulation and hence β_2_-adrenoceptor-mediated signaling, does not appear to be related to the proliferative capacity of the clonal populations.

### Altered Gq-coupled receptor signaling in human ASM clonal populations

To assess pro-contractile Gq-coupled receptor-mediated signaling, bradykinin and histamine were utilised to induce a concentration-dependent accumulation of [^3^H] Inositol Phosphate (IP) in human ASM clonal cell populations (Figure 6C & D). The highest [^3^H] IP was produced by the unsorted, donor-matched ‘standard’ cell type in response to stimulation with the maximum dose of the contractile agonists namely bradykinin (10 μM) (5.16 ± 0.4 fold increase cf vehicle, Figure 6C) and histamine (100 μM) (2.63 ± 0.3 fold increase cf vehicle Figure 6D). As can be observed in Figure 6C exposure to bradykinin was observed to induce the maximum observed increase in [^3^H] IP in the Slow Growing clonal populations (2.9 ± 0.2 fold increase cf vehicle) compared to the Fast Growing clones (2.78 ± 0.2 fold increase cf vehicle). The same trend was observed following histamine stimulation (Figure 6D) with the slow proliferating clones demonstrating higher levels of [^3^H] IP (2.17 ± 0.04 fold increase cf vehicle) than the Fast Growing populations (1.98 ± 0.2). However these differences were not statistically significant. Statistical analyses of the data across all the clones following bradykinin and histamine stimulation showed a significant difference in the production of [^3^H] IP (p < 0.001 and p = 0.0023 by two-way ANOVA respectively).

## Discussion

This is the first study to examine the clonogenic potential of human ASM cells. The main objective of this study was to determine whether different lineages of airway mesenchymal cells can be derived from early passage cultures of human ASM cells by single-cell cloning. The next aim was to investigate their morphological, phenotypic and functional characteristics. Critically, our studies demonstrate that the majority of single cells present within early passage human ASM cell cultures have the potential to create expanded cell populations. The clonal populations exhibited dramatic differences in their doubling times ranging from 1.1 to 18.3 days in the 58% of clones which proliferated sufficiently to be quantified. Furthermore morphological and functional heterogeneity were also seen to exist within these clonal populations. These findings confirm the existence of a heterogeneous group of cells within these cultures which may have the potential to develop diverse phenotypes (hypercontractile, contractile or synthetic). Our observation that the majority of cells isolated from early passage cultures of human ASM are capable of expansion from a single isolated cell indicates the presence of large numbers of cells with significant proliferative potential in this cell population.

Functional data from our studies demonstrate varied proliferative response by different human ASM clones, both in the absence of mitogens and also following stimulation with PDGF. In our study these clones were classified into groups based on their doubling time in the absence of the mitogens. Interestingly, the fast-growing clones did not only have higher basal proliferation rates but also exhibited a higher proliferative response following PDGF stimulation suggesting these cells have an intrinsic pro-proliferative phenotype.

Based on previously published studies [18-23] we hypothesized that the majority of the cells within the fast-growing clonal population would be of a “synthetic” phenotype and the slow-growing clonal populations would comprise more of the “contractile” forms. Hence we attempted to look at the phenotype characteristics of these two different clonal populations by immunocytochemistry. We used a range of previously published markers [11] to identify human ASM cells (the classical contractile form) and human airway fibroblasts (the classical synthetic phenotype). We have previously reported the overlap between ASM and fibroblasts and from this study chose to use α-smooth muscle actin, FSP, cathepsin K, thromboxane synthase and α8 integrin [11]. Contrary to expectation, the fast growing clonal population had, if anything, slightly increased levels of α smooth muscle actin and the slow growing populations had increased levels of FSP. It is important to recognise that both the standard culturing conditions used and the specific conditions required for the different experiments each have the capacity to promote different phenotypes. For example culturing with serum (as was done through the clonal expansion phase of the study) would be expected to promote the synthetic phenotype whereas the serum deprivation performed prior to immunofluorescent and second messenger studies should promote an increased proportion of cells exhibiting contractile characteristics. These experimental conditions are largely unavoidable i.e. attempting to derive clonal populations from single cells is unlikely to be successful. Another consideration is how the stiff plastic substrate on which all cells were cultured and on which experiments were performed itself promotes the enrichment of certain phenotypes. Whilst it is widely recognized that extracellular matrix composition alters ASM phenotype and signaling capabilities [10,20,24], it is increasingly being recognised that the physical properties of the substrate alone can also affect phenotype i.e. static vs dynamic, 2D vs 3D, differential stiffness as recently reviewed [25]. Whether the expansion of clonal populations in a more physiological environment would result in different outcomes to those reported here is unknown.

The coexistence of cell populations with diverse proliferative responses is key in understanding the relationship between ASM cell heterogeneity and airway remodeling. It is important to remember that the cells described in this manuscript represent a population which has been effectively enriched for proliferative properties with non-proliferative cells being lost following the original isolation of the ASM cells and early passaging. However, that there remains such cellular diversity in phenotype from a culture of cells which have already been through two passages is still perhaps surprising. So what could be the origin of the observed populations? And why would a clonally-derived population of cells exhibit diverse morphological differences in identical culture conditions? Possible sources of these highly proliferative populations are mesenchymal stem cells or progenitor cells such as fibrocytes either from peripheral blood or from within the tissue [6,15]. To date it is unclear whether mesenchymal stem cell populations exist within airway smooth muscle cell bundles and are able to contribute to the increased smooth muscle mass observed in asthma [6]. A little more is understood about lung fibrocytes which are fibroblast-like progenitor cells first detected outside the circulation in human bronchial mucosa in patients with chronic allergic asthma [26]. Intriguingly, fibrocytes exposed to TGFβ have been observed to become more smooth muscle-like in phenotype [27]. In addition, levels of fibrocytes have been correlated with the yearly decline in lung function in patients with asthma presenting with chronic airflow obstruction [27]. Recently the same group reported an increase in the accumulation of fibrocytes in bronchial walls of patients with the same clinical phenotype [28]. This is in agreement with the study by Saunders et al., where elevated number of fibrocytes were observed in the ASM bundles of individuals with asthma compared with controls, however there was no correlation between this and lung function [29]. Another possible source of myofibroblasts into the airways is via epithelial-mesenchymal transitions, however emerging evidence from lineage-tracing studies raises the possibility that the latter may just be a consequence of in vitro culture and not occur *in vivo*[30].

It is however important to note that the cultures used for the experiments we describe here were derived from nonasthmatic human airways and hence would be unlikely to contain fibrocyte-derived populations if such cells only traffic to the lung following airway inflammation.

It was interesting to note that the proliferative potential of each clonal population did not appear to influence either (pro-relaxant) cyclic AMP-mediated or (pro-contractile) IP3-mediated signaling, especially when considered with the observation that cells with an increased proliferative rate exhibited increased α-smooth muscle actin and decreased FSP expression. These data suggest that it is perhaps not feasible to dichotomize the ASM populations into “good” (anti-proliferative, anti-contractile, pro-relaxant) and “bad” (being the opposite) as might have been assumed from previous studies where a proliferative, synthetic phenotype is associated with reduced contractile protein [18-23]. However, as evidenced by the diverse morphology observed within clonal populations (which we did not address quantitatively), there are subpopulations of cells within the clonal populations which are quite different. So whilst we observed differences *between* the clonal populations, it would be revealing to understand how subpopulations *within* these further differ, particularly in terms of GPCR density or signaling capacity. Seminal studies in this field from Halayko and colleagues using canine tracheal smooth muscle cells both characterized subpopulations which arose after prolonged serum deprivation [31]. Here one sixth of the cells were observed to exhibit a contractile phenotype as characterized by elongated morphology, alignment into bundles, increased expression of smooth muscle α-actin, smooth muscle myosin heavy chain and SM22 and, importantly, expression of muscarinic M3 receptors which are usually lost from smooth muscle cells through culturing [31]. Thus whether subpopulations within the clonal populations exhibited diverse GPCR expression remains to be explored.

## Conclusions

In summary, we have shown that human ASM cell cultures represent heterogeneous cell populations that exhibit morphological and functional differences. Our studies also reveal that a significant proportion of individual early passage human ASM cells have the potential to create expanded cell populations suggesting the presence of large number of mesenchymal progenitors. Taken together we suggest that these phenotypically diverse cell populations existing within ASM cultures may contribute differentially to inflammatory and remodelling processes within the airways.

## Consent

Written informed consent was obtained from the patient for the publication of this report and any accompanying images.

## Abbreviations

ASM: Airway smooth muscle; cyclic AMP: Cyclic adenosine monophosphate; FACS: Flow activated cell sorting; FCS: Foetal calf serum; PDGF: Platelet derived growth factor; TGF-β: Transforming growth factor-beta; AKR1C3: Aldo-keto reductase 1 C3; TBXAS1: Thromboxane synthase 1; PGE2: Prostaglandin E2.

## Competing interests

The authors declare that they have no competing interests.

## Authors’ contributions

SRS conducted the experiments, participated in the design and analysis of the study and drafted the manuscript. CKB assisted with the clonal expansion studies, participated in the design and analysis of the study and drafted the manuscript. IS critically reviewed the manuscript. IPH conceived of the study, participated in its design and coordination and helped to draft the manuscript. All authors read and approved the final manuscript.
